# Molecular Clone and Expression of a NAD^+^-Dependent Glycerol-3-Phosphate Dehydrogenase Isozyme Gene from the Halotolerant alga *Dunaliella salina*

**DOI:** 10.1371/journal.pone.0062287

**Published:** 2013-04-23

**Authors:** Ma Cai, Li-Hong He, Tu-Yuan Yu

**Affiliations:** College of Life Sciences, Zhongkai University of Agriculture and Engineering, Guangzhou, China; St. Georges University of London, United Kingdom

## Abstract

Glycerol is an important osmotically compatible solute in *Dunaliella*. Glycerol-3-phosphate dehydrogenase (G3PDH) is a key enzyme in the pathway of glycerol synthesis, which converts dihydroxyacetone phosphate (DHAP) to glycerol-3-phosphate. Generally, the glycerol-DHAP cycle pathway, which is driven by G3PDH, is considered as the rate-limiting enzyme to regulate the glycerol level under osmotic shocks. Considering the peculiarity in osmoregulation, the cDNA of a NAD^+^-dependent *G3PDH* was isolated from *D. salina* using RACE and RT-PCR approaches in this study. Results indicated that the length of the cDNA sequence of *G3PDH* was 2,100 bp encoding a 699 amino acid deduced polypeptide whose computational molecular weight was 76.6 kDa. Conserved domain analysis revealed that the G3PDH protein has two independent functional domains, SerB and G3PDH domains. It was predicted that the G3PDH was a nonsecretory protein and may be located in the chloroplast of *D. salina*. Phylogenetic analysis demonstrated that the *D. salina* G3PDH had a closer relationship with the G3PDHs from the *Dunaliella* genus than with those from other species. In addition, the cDNA was subsequently subcloned in the pET-32a(+) vector and was transformed into *E. coli* strain BL21 (DE3), a expression protein with 100 kDa was identified, which was consistent with the theoretical value.

## Introduction

*Dunaliella salina*, one member of the genus *Dunaliella* (Chlorophyceae, Volvocales), is an extremely halotolerant, unicellular, green, and motile algae. The genus *Dunaliella* is unique in its remarkable ability to survive in the media with a wide range of NaCl concentrations, from about 0.05 M to saturation (around 5.5 M), while maintaining a relatively low intracellular sodium concentration [1], [2]. This remarkable osmotic adaptability is mediated primarily by the massive *de novo* synthesis of the compatible solute, the glycerol, following salt stress [3]. These characteristics make *D. salina* an obvious applicable value as a model organism in studying the mechanism of osmoregulation under salt environment conditions. In addition, under high salt stress, *D. salina* could accumulate large amounts of β-carotene in cells, which makes it one of the best sources of natural β-carotene [4]–[7].

Glycerol is an important osmotically compatible solute in *Dunaliella* under salt stress [8], [9]. The extracellular osmotic pressure is released in Dunali*ella* by changing intracellular glycerol content. The glycerol is synthesized rapidly when the concentration of saline increases, and the glycerol transforms to starch when the concentration of saline drops [10]–[12]. At high salinity, *D. salina* accumulates massive amounts of glycerol and the level of intracellular glycerol is proportional and osmotically equivalent to the external NaCl concentration, reaching about 8 M or 55% (w/v) of the cell weight at saturated NaCl concentrations [13], . Moreover, the green alga *Dunaliella tertiolecta* could also adapt the different concentration of saline by synthesizing or eliminating the intracellular glycerol to balance the osmotic potential of intracellular and extracellular [15], . Nicotinamide adenine dinucleotide (NAD^+^)-dependent glycerol-3-phosphate dehydrogenase (G3PDH) plays a major role in the osmoregulation process in *Dunaliella* [12], [17]. In glycerol biosynthesis pathway, G3PDH catalyze dihydroxyacetone phosphate (DHAP) to form glycerol-3-phosphate, which is converted to glycerol finally by glycerol-3-phosphate phosphatase [18], [19].

It was found that there are five isozymes of G3PDH in *D. salina*, and these isozymes respectively take effects in different salinities and play important roles in glycerol metabolism [12]. Chen et al found that four loci produced different G3PDH isozymes functioning under different salinity conditions [12]. In this study, we isolated the cDNA of a NAD^+^-dependent G3PDH from *D. salina*, which is one isozyme with highly homology of previously isolated G3PDH in this alga [20]. Subsequently, a series of bioinformatics tools were employed for the analysis of its physical-chemical characteristic, conserved structural domain, transmembrane and signal sequence condition, secondary and spatial structure, phylogenesis, and so on. Finally, this G3PDH was subcloned in the pET-32a(+) vector and undergone prokaryotic expression to further elucidate the pathway of glycerol metabolisms in *Dunaliella*.

## Materials and Methods

### Cultivation of *D. salina* under Salt Stresses

Cells of *D. salina* strain 435 (UTEX 200) conserved in our laboratory were cultivated in the culture medium according to Chen et al [21]. Cells grown at the late log phase were harvested by centrifugation at 5,000 g for 15 min at 4°C for next experimental procedure.

### Isolation of cDNA for G3PDH in *D. salina*

The total RNA was prepared from 10 mL of *D. salina* cells grown at the late log phase with using E.Z.N.A. Total RNA Kit II (OMEGA) according to the manufacture’s instruction. Subsequently, the total RNA was treated by DNase I (RNase Free) (TaKaRa) and was dissolved in 0.1% (v/v) diethyl pyrocarbonate solution (TaKaRa) [7], [22].

The first strand of cDNA was synthesized from the total RNA using PrimeScript ™ RT-PCR kit (TAKARA) according to the manufacturer’s instructions [7], [22]. Reverse transcription (RT) reaction was performed with the parameters set as follows: 42°C for 30 min, followed by 70°C for 15 min. Primers Dsgpdh1-F and Dsgpdh1-R were used to amplify the conserved fragment of the *D. salina* G3PDH cDNA by using Premix Ex Taq (TaKaRa) following the manufacturer’s instructions. The PCR procedure is as the following: 1 cycle of 94°C, 5 min; 30 cycles of 94°C, 30 s, 51°C, 30 s, and 72°C, 1 min; and 1 cycle of 72°C, 10 min; used primers listed in Table 1.

**Table 1:** Primers used in this study (5′-3′).

Procedure	Primer	Primer sequence (5→3)
ESTisolation	Dsgpdh1-F	CAACGAGAACCATGAGAACC
	Dsgpdh1-R	CACTGAGGGGGAGATGAACTTGC
3'RACE	Dsgpdh3'F	CAATGTCGCCAGCAATGTTA
5'RACE	Dsgpdh5'F1	AACTGAACTTCACCCCCACAGACAT
	Dsgpdh5’F2	GGCTCGTGGAGTGGAGGTGT
cDNAisolation	Dsgpdh-F	TTAGTAGTAGTCGTTCACTACACGG
	Dsgpdh-R	ATGCTTCTCCAGAAAGGAAACATTG
ORFsubclone	ORF-F	CGGGATCCATGCTTCTCCAGAAAGGAAAC
	ORF-R	CCCTCGAGTTAGTAGTAGTCGTTCACTACACG

Based on the obtained conserved cDNA fragment sequence, gene specific primer Dsgpdh3’F was designed and 3′ RACE was conducted with oligo dT-Adaptor primers using RNA PCR Kit (AMV) Ver.3.0 (TaKaRa). The first-strand cDNA was amplified by LA Taq (TaKaRa) with the parameters set as follows: 94°C, 4 min; 30 cycles of 94°C, 30 s, 46°C, 30 s, and 72°C, 1 min with a final extension at 72°C for 10 min.

The 5′ RACE operation was accomplished with SMART™ MMLV Reverse Transcriptase (Clontech) and synthesized primers SMARTAO and 5′-RACE CDS. The second 5′RACE was conducted using the Dsgpdh5’F2primer designed according to the fragment obtained from the first 5′RACE reaction with Dsgpdh5’F1 primer. Other handlings including touchdown PCR were employed according to the SMARTer™ RACE cDNA Amplifcation Kit User Manual, with the exception of LA Taq DNA polymerase (TaKaRa) for touchdown PCR, rather than Advantage 2 Polymerase Mix.

The full-length G3PDH cDNA was obtained with specific primes (Dsgpdh-F and Dsgpdh-R, Table 1) corresponding to the 5′ and 3′ ends of the G3PDH gene. The PCR procedure to amplify the G3PDH cDNA fragment is as follows: 1 cycle of 94°C, 5 min; 30 cycles of 94°C, 30 s, 55°C, 30 s, and 72°C, 1 min; and 1 cycle of 72°C, 10 min. All amplified fragments were cloned into pCR2.1 vector (Invitrogen) and undergone Sanger sequencing.

All PCR products were separated by electrophoresis in 1.5% (w/v) agar gels, cloned in the pMD18-T vector (TaKaRa) and sequenced before the further experiments. Plasmid preparations, transformations, and other standard molecular biology techniques were carried out as described previously [23].

### Bioinformatics Analysis and Phylogenetic Construction

Sequence analysis was performed using Blast Software (http://blast.ncbi.nlm.nih.gov/). Component analysis of G3PDH was calculated using DNAStar software 7.1.0 (Lasergene). Physical and chemical characteristics of G3PDH were analyzed by ProtParam tool (http://www.expasy.ch/tools/protparam.html). Multiple alignments among similar enzymes were conducted using Clustal X 1.83 (NCBI, Bethesda, MD). Phylogenetic and molecular evolutionary analysis of the amino acid sequences of different G3PDHs were conducted using the Neighbor Joining method and the molecular evolution genetics analysis (MEGA) software, version 4.0.2. The conserved structural domain of G3PDH was predicted via National Center for Biotechnology Information conserved domain database (CDD) in NCBI (http://www.ncbi.nlm.nih.gov/Structure/cdd/wrpsb.cgi), and the protein conserved module of G3PDH was analyzed via the Pfam database (http://pfam.sanger.ac.uk/) to search its domain combinations. The amino acid sequence was subjected to TMHMM server (http://www.cbs.dtu.dk/services/TMHMM/) for transmembrane analysis and SignalP 3.0 Server (http://www.cbs.dtu.dk/services/SignalP/) for the prediction of protein signal sequence. Subcellular localization presumption was performed using WoLF PSORT (http://wolfpsort.org/). Secondary structure was predicted via the NPS@ service (http://npsa-pbil.ibcp.fr/) and PredictProtein (https://www.predictprotein.org/); 3D structure was constructed using 3D-JIGSAW (http://bmm.cancerresearchuk.org/~3djigsaw/).

### Plasmid Construction and Protein Expression

Plasmid pET-32a-G3pdh was constructed by insertion of the *D. salina* G3PDH open reading frame (ORF) into the *Bam*H I and *Xho* I (all restriction endonucleases are products of TaKaRa, Japan) restriction sites of expression vector pET-32a(+) (Novagen, Darmstadt, Germany). The G3PDH cDNA fragment was used as templates to synthesize the G3PDH ORF by using PrimSTAR HS DNA Taq (TaKaRa, Japan) with forward primer ORF-F and reverse primer ORF-R, which will introduce the *Bam*H I and *Xho* I site into the 5′ and 3′ end of the ORF, respectively. The PCR procedure is as the following: 1 cycle of 94°C, 30 s; 30 cycles of 94°C, 30 s, 60°C, 30 s, and 72°C, 2 min; and 1 cycle of 72°C, 10 min. PCR product was separated by electrophoresis in 1.5% (w/v) agar gels, cloned in the pMD18-T vector and transformed into *E. coli* JM109, which was verified by double digestion of *Bam*H I and *Xho* I. Both plasmids pET-32a and pMD18-T-JM109-G3pdh were prepared from *E. coli* BL21 and JM109, then digested by *Bam*H I and *Xho* I at 30°C for 45 min or 2 h. The digested products were separated by electrophoresis in 1% (w/v) and 1.5% (w/v) agar gels, and the ORF and pET-32a fragments were recycled using E.Z.N.A. kit (OMEGA, USA). T4 DNA ligase (0.5 µL containing 200 NEB units; New England Biolabs, USA) was then added, and samples were incubated at 16°C for 12–16 h. 5 µL of the ligation mixture was used to transform electronically competent *E. coli* (100 µL of BL21(DE3)). The correct strain of *E. coli* BL21 (DE3)/pET-32a-G3pdh was verified by double digestion of *Bam*H I and *Xho* I.

*E. coli* strains BL21 (DE3) were grown in LB medium at 37°C in darkness on a platform shaker at 230 cycles min^−1^. Ampicillin (100 µg mL^−1^) was used for selection or maintenance of plasmids. To induce the expression of the *D. salina* G3PDH, a final concentration of 1.0 mmol L^−1^ isopropyl-β-D-thiogalactopyranoside (IPTG) was added to the *E. coli* culture when the optical density (OD) value reached 0.4–0.6, and the culture was allowed to continue growing for 3–4 h before harvesting by centrifugation.

### SDS-PAGE Electrophoresis Analysis

The transformed cells with plasmid pET-32a-G3pdh or pET-32a was disrupted by ultrasonication in PBS buffer containing 1 mmol L^−1^ phenylmethysulfonyl fluoride (PMSF). The supernatants were collected by centrifugation at 12,000 *g* for 30 min at 4°C. Protein concentration was estimated as described [24]. Then supernatant in each samples were boiled for 5 min after adding 5×SDS loading butter (4:1, v:v); protein molecular weight marker was also boiled for 3 min before loading samples. 10 µg of each samples were subjected to SDS-PAGE in a Bio-Rad protein electrophoresis system (Bio-Rad, Hercules, CA, USA), which was carried out at 80 V, and increased to 120 V after 1 h. The concentration of separating gel and stacking gel were 12% (w/v) and 5% (w/v). Protein electrophoretic profiles in gels were visualized through Coomassie Blue R-250 staining procedure.

## Results

### Isolation of cDNA for G3PDH from *D. salina*

**Figure 1:**
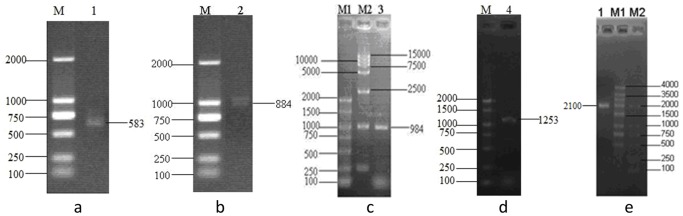
Isolation of *D.salina* G3PDH gene fragments. (a)EST fragment of 583 bp. M, DNA marker; lane 1, PCR product. (b)3′ RACE fragment of the 884 bp. M, DNA marker; lane 2, PCR product; (c)First 5′ RACE fragment of 984 bp. M1, M2, DNA marker; lane 3, PCR product. (d)Second 5′ RACE fragment of 1253 bp. M, DNA marker; lane 4, PCR product. (e) The full-length cDNA of *D. salina*.

A pair of specific primers were designed to obtain G3PDH cDNA conserved fragment from *D. salina* on the basis of the G3PDH gene sequences of *D. salina*, *Chlamydomonas reinhardii*, *Arabidopsis thaliana*, *Pichia stipitis*, *zygosaccha* and four predicted G3PDH gene sequences. When using total RNA from *D. salina* cells as RT substrate, an expected 583 bp fragment amplified with primers Dsgdph1-F/R was cloned and sequenced (Fig. 1a).

**Figure 2:**
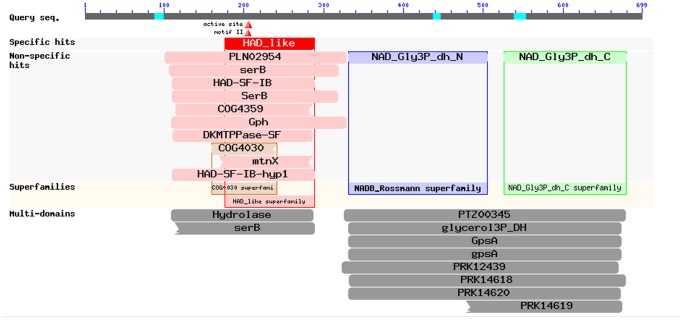
Conserved domains in G3PDH detected by NCBI Conserved Domains Search. The deduced 699-amino-acid sequence is used to search. Three conserved regions including phosphoserine phosphatase (SerB) domain, N- and C- terminals binding to NAD^+^ were predicted.

On the basis of this cDNA conserved fragment, the 3′ end fragment was amplified by 3′ RACE reaction, which was 884 bp in length (Fig. 1b); and then two 5′ RACE reactions were fulfilled resulting two fragments with 984 bp in the first step (Fig. 1c) and 1253 bp in the second step (Fig. 1d), respectively. Then, based on the sequence assembly, full-length G3PDH cDNA was amplified using specific primers Dsgpdh-F/R, which was 2100 bp (Fig. 2).

### Bioinformatics Analysis of G3PDH cDNA and Amino Acid Sequence

Nucleotide sequence analysis showed that the *D. salina* G3PDH cDNA contained 2100 bp nucleotides with an ORF of 2100 bp, which contained 19.29% A, 31.48% G, 29.05% C and 20.18% T. The ORF encoded a 699-amino-acid-long peptide including 78 basic amino acids (lysine, arginine), 83 acidic amino acids (aspartic acid, glumatic acid), 266 hydrophobic amino acids (isoleucine, leucine, phenylalanine, tryptophan, valine) and 144 polar amino acids (Asparagine, cysteine, serine, threonine, tyrosine). Analysis by ProtParam tool revealed that the molecular weight of this peptide was 76.6 kDa, the isoelectric point was 6.49.

A complete homologous search by BLAST demonstrated that the nucleotide and putative protein sequence had, respectively, sequence identities of 91% and 95% with the published *D. salina* G3PDH with NAD^+^ as coenzyme (AY845323.1), 83% and 78% with *D. viridis* G3PDH1 (EU624406.1), 76% and 72% with *D. viridis* G3PDH2 (EU624407.1), which indicated that the protein encoded by the obtained cDNA in this study might belong to the G3PDH family with NAD^+^ as coenzyme.

**Figure 3:**
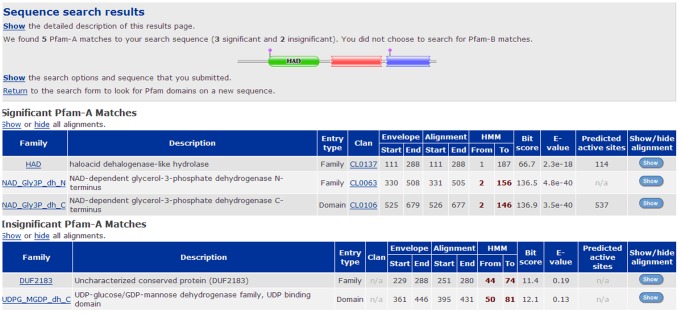
Conservative module analysis of G3PDH by Pfam. Similar with CDD search, three conserved domains were found; with the exception that SerB domain was predicted to be affiliated to similar hydrolase domain family.

The Conserved Domain Database (CDD) provided by NCBI was employed to predict the structural and functional region (Fig. 2), which manifested that the putative polypeptide from *D. salina* contained three conserved regions including phosphoserine phosphatase (SerB) domain, N- and C- terminals binding to NAD^+^ (Fig. 2). Using Pfam database to search and predict the structural and functional domain of this putative polypeptide gained a similar result as shown by Fig. 3. The difference from CDD conservative structure speculated by NCBI was that SerB domain was affiliated to similar hydrolase domain family. The point also elucidated the first 330 amino acids should possess hydrolase function, and the latter amino acids played the main role of glycerol-3-phosphate dehydrogenase. Consequently, the results predicted that this deduced protein was a NAD^+^-dependent G3PDH (EC 1.1.1.8). Eukaryotic neural network (NN) search by SignalP 3.0 showed that this polypeptide had no signal peptide. It was also predicted to be non-secretory protein by Markov models (HMM) of SignalP 3.0. TMHMM Server v. 2.0 predicted that the G3PDH had no transmembrane domain. Presumption of subcellular localization performed using WoLF PSORT showed that *D. salina* G3PDH may be situated in the chloroplast.

### Prediction of Protein Structure

The secondary structure of the protein was deduced by NPS@service PHD, GOR1, SOPMA and PREDATOR methods. On account of the distinct emphases of various methods, the predicted results were also different. So these methods were made a comparison and PredictProtein was adopted to analyze online (Table 2). As shown by Table 2, the G3PDH protein had abundant α-helixes, some extended strands, many random coils and a few β-turns, but had no 3_10_-helix, π-helix and other rare secondary structure.

**Table 2:** Prediction of protein secondary structure of *D. salina* G3PDH.

	α-helix	Extendedstrand	Randomcoil	β-turn
PHD	45.21%	8.15%	46.64%	0%
GOR1	52.79%	32.76%	7.73%	6.72%
SOPMA	46.35%	13.45%	32.05%	8.15%
PREDATOR	36.48%	10.73%	52.79%	0%
PredictProtein	45.21%	12.45%	42.35%	0%

**Figure 4:**
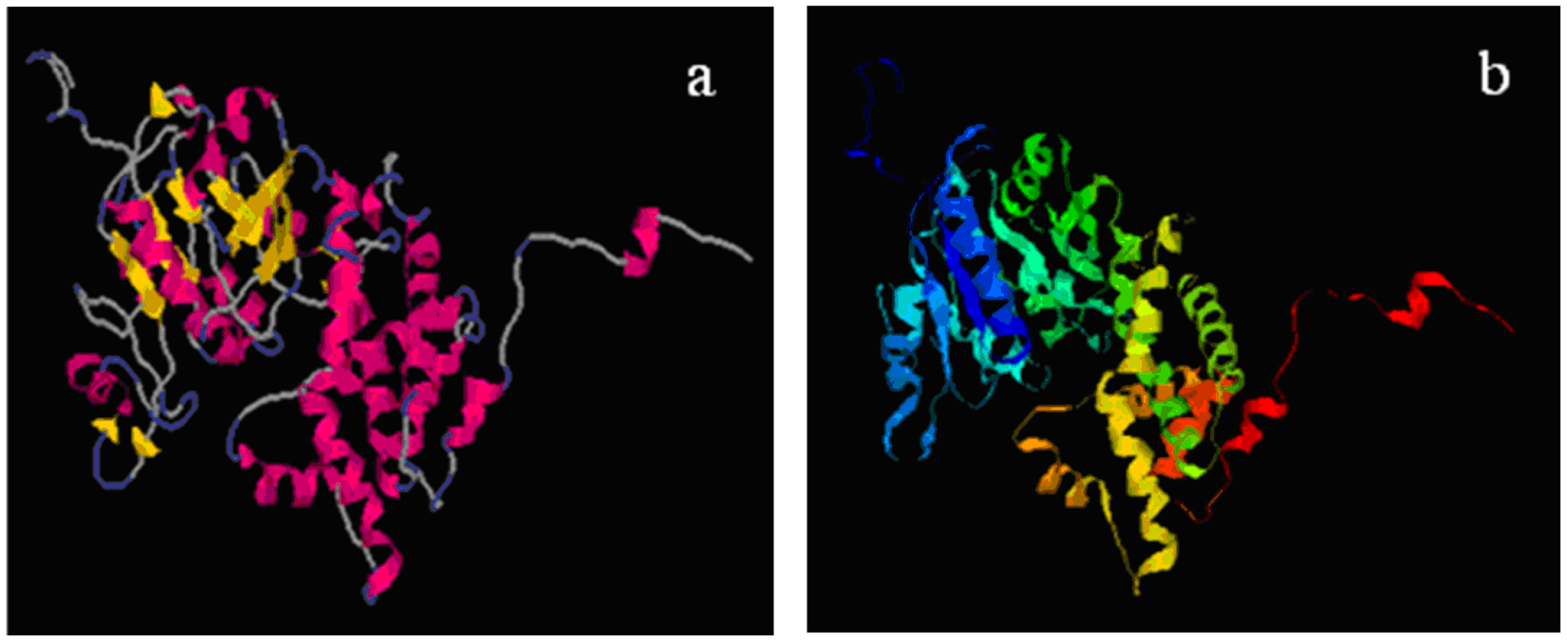
Schematic representation of three-dimensional models of the *D.salina* G3PDH. (a) Comparative modeling was performed using 3D-JIGSAW. (b) Comparative modeling was performed using CPH models. The α-helix and β-sheet regions of the putative protein are indicated with ribbons and arrows, respectively. The loop regions are also designated in the schematics.

Three-dimensional structure of the *D. salina* G3PDH was predicted by 3D-JIGSAW comparative modeling program based on homologues of known structures automatically, and was visualized using RasMol software (Fig. 4). A big gap could be observed in the center of the protein by Fig. 4 using both the cartoon and ribbon models, which was regarded as the possible active site where G3PDH reacted with substrate and avoided external interference due to the protection of substrate.

### Multiple Sequence Alignment and Phylogenetic Analysis

**Figure 5:**
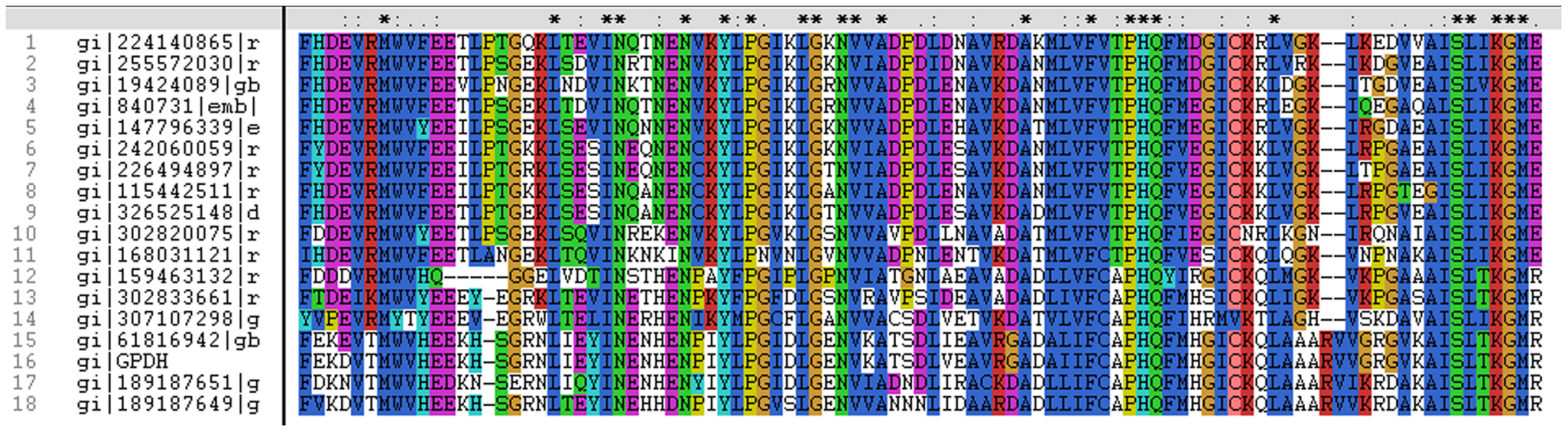
Alignment of ***D.salina*** G3PDH with G3PDHs sequences from other species. GPDH: G3PDH in the present study. Other G3PDHs are shown as GenBank accession number: *D. salina* G3PDH (GI 61816942), *D. viridis* G3PDH (GI 189187651), *D. viridis* G3PDH (GI 189187649), *Chlamydomonas reinhardtii* predicted G3PDH (GI 159463132), *Chlorella variabilis* hypothetical G3PDH (GI 307107298), *Volvox carteri* hypothetical G3PDH (GI 302833661), *Hordeum vulgare* predicted G3PDH (GI 326525148), *Sorghum bicolor* hypothetical G3PDH (GI 242060059), *Zea mays* G3PDH (GI 226494897), *Oryza sativa* Japonica Group G3PDH (GI 115442511), *Cuphea lanceolata* G3PDH (GI 840731), *Vitis vinifera* hypothetical G3PDH (GI 147796339), *Arabidopsis thaliana* putative G3PDH (GI 19424089), *Populus trichocarpa* predicted G3PDH (GI 224140865), *Ricinus communis* putative G3PDH (GI 255572030), *Selaginella moellendorffii* hypothetical G3PDH (GI 302820075), *Physcomitrella patens subsp. patens* predicted G3PDH (GI 168031121).

The deduced amino acid sequence of G3PDH was multiple sequences aligned with those from different species by Clustal X1.8. These sequences and Genbank accession numbers are as the following: *D. salina* G3PDH (GI 61816942), *D. viridis* G3PDH (GI 189187651), *D. viridis* G3PDH (GI 189187649), *Chlamydomonas reinhardtii* predicted G3PDH (GI 159463132), *Chlorella variabilis* hypothetical G3PDH (GI 307107298), *Volvox carteri* hypothetical G3PDH (GI 302833661), *Hordeum vulgare* predicted G3PDH (GI 326525148), *Sorghum bicolor* hypothetical G3PDH (GI 242060059), *Zea mays* G3PDH (GI 226494897), *Oryza sativa* Japonica Group G3PDH (GI 115442511), *Cuphea lanceolata* G3PDH (GI 840731), *Vitis vinifera* hypothetical G3PDH (GI 147796339), *Arabidopsis thaliana* putative G3PDH (GI 19424089), *Populus trichocarpa* predicted G3PDH (GI 224140865), *Ricinus communis* putative G3PDH (GI 255572030), *Selaginella moellendorffii* hypothetical G3PDH (GI 302820075), *Physcomitrella patens subsp. patens* predicted G3PDH (GI 168031121). The results indicated the general conservatism of botanical and algal G3PDH genes (Fig. 5). Consequently, according to the location of the conservative region, the functional region of the G3PDH presumably started at the 331st amino acid of the sequence and the 330 amino acids prior to it possibly involved in other properties of protein, which had explanation in structural and functional predication section above.

**Figure 6:**
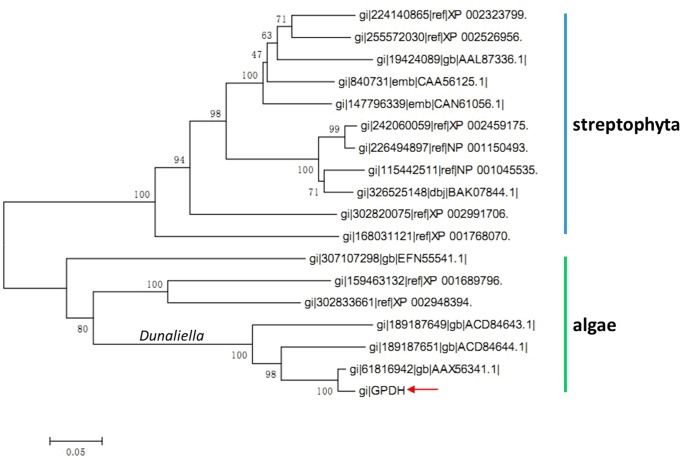
Phylogenetic tree (neighbor-joining) for G3PDH of 18 species. *D. salina* G3PDH (GI 61816942), *D. viridis* G3PDH (GI 189187651), *D. viridis* G3PDH (GI 189187649), *Chlamydomonas reinhardtii* predicted G3PDH (GI 159463132), *Chlorella variabilis* hypothetical G3PDH (GI 307107298), *Volvox carteri* hypothetical G3PDH (GI 302833661), *Hordeum vulgare* predicted G3PDH (GI 326525148), *Sorghum bicolor* hypothetical G3PDH (GI 242060059), *Zea mays* G3PDH (GI 226494897), *Oryza sativa* Japonica Group G3PDH (GI 115442511), *Cuphea lanceolata* G3PDH (GI 840731), *Vitis vinifera* hypothetical G3PDH (GI 147796339), *Arabidopsis thaliana* putative G3PDH (GI 19424089), *Populus trichocarpa* predicted G3PDH (GI 224140865), *Ricinus communis* putative G3PDH (GI 255572030), *Selaginella moellendorffii* hypothetical G3PDH (GI 302820075), *Physcomitrella patens subsp. patens* predicted G3PDH (GI 168031121).

The phylogenetic tree for the complete homologous G3PDHs was constructed using neighbor-joining method by MEGA 4.0.2 software. As Figure 6 shown, the G3PDH obtained in this study (indicated by red arrow in Fig. 6) share highest evolutionary position with other homologues from the *Dunaliella* genus, they all clustered into the green algae group with *Chlamydomonas reinhardtii* and *Chlorella variabilis*.

### Prokaryotic Expression, Protein Purification and Enzymatic Assay

**Figure 7:**
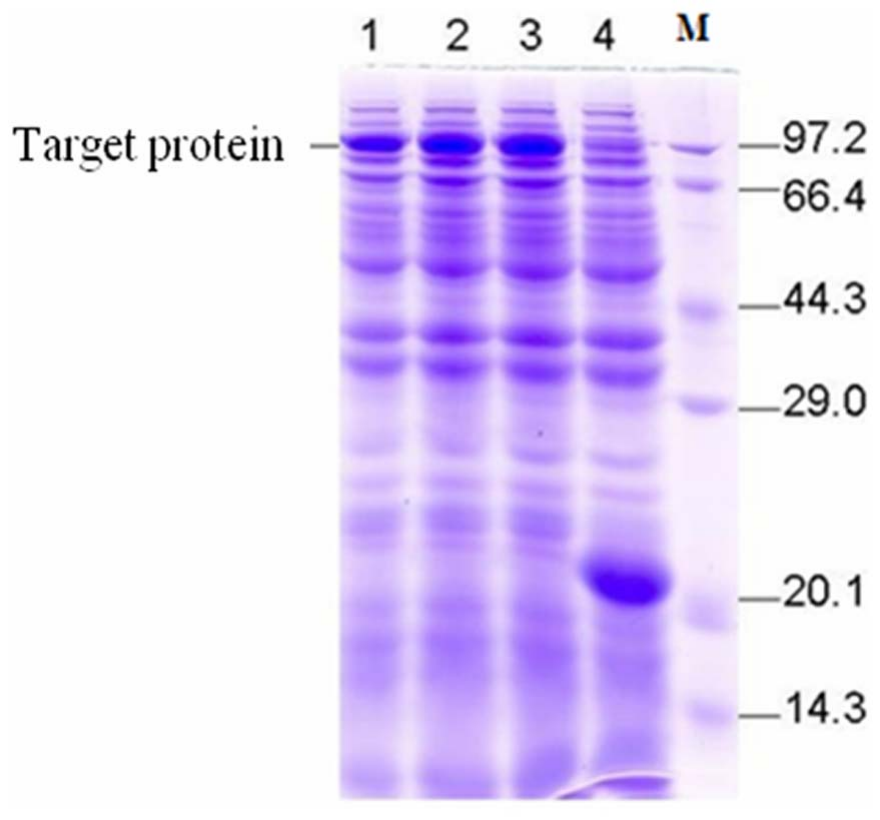
10% (w/v) SDS-PAGE of expression of interest protein in *E.coli*. M, protein marker; lane 1, 2 and 3, protein sample with loading volume of 10, 15 and 20 µl, respectively; lane 4, negative control (empty vector control, pET-32a).

The G3PDH ORF sequence of 2100 bp was amplified by PCR with *D. salina* cDNA as templates. The cDNA was subsequently subcloned in the pET-32a(+) expression vector in the *Bam*H I/*Xho* I sites. The constructed prokaryotic expression vector pET-32a-G3pdh was transformed into *E. coli* strain BL21 (DE3) and IPTG was used for induction. The G3PDH in transgenic strains were analyzed by SDS-PAGE. As shown by Fig. 7, a clear protein expression band could be observed at the position of 100 kDa (the addition of target gene 76.6 kDa and histidine marker 23 kDa), which was consistent with the theoretical value. Whereas, an obvious protein expression band appeared at the location of 20 kDa in positive control and no band was found in the samples due to the inhibition of the protein expression by the introduced target gene (Fig. 7).

## Discussion

As is known, the osmotic adjustment response of *Dunaliella* (especially *D. salina*) functioned by varying the intracellular concentration of a compatible solute glycerol to balance the osmotic pressure inside and outside of cells. When subjected to hyperosmotic shock *D. salina* cells rapidly shrink followed by synthesis of glycerol to increases the internal osmolarity for resuming original volume of cells. Under hypoosmotic shock, it was found rapid swells followed by a decrease in internal glycerol and volume resumption [21]. The biosynthesis of glycerol in *D. salina* involved the key enzymes G3PDH, which convert DHAP to glycerol-3-phosphate. Some studies have been performed to investigate the G3PDH for elucidating the mechanism of osmotic stress tolerance by glycerol. In a study, a novel G3PDH (NAD^+^) (EC1.1.1.8) gene (*PfGPD*) was cloned from halotolerant yeast *Pichia farinosa*, and the *PfGPD* gene was induced by salt stress [25]. In another study, He et al cloned the cDNA encoding a NAD^+^-dependent G3PDH from *D. salina*, and the cDNA may encode an osmoregulated isoform primarily involved in glycerol synthesis [20]. In addition, He et al have cloned two novel chloroplastic G3PDH cDNAs (*DvGPDH1* and *DvGPDH2*) from *Dunaliella viridis*, which encode two polypeptides of 695 and 701 amino acids, respectively [26]. Q-PCR analysis revealed that both genes exhibited transient transcriptional induction of gene expression upon hypersalinity shock, followed by a negative feedback of gene expression.

In the present study, the cloned 2100 bp G3PDH cDNA from *D. salina* acts with NAD^+^ as coenzyme, and the comparative study of conservative regions discovered NAD^+^ and 3-phosphoglycerate binding sites in the G3PDH protein, theoretically testifying the cloned cDNA encodes G3PDH of osmosis-adjusting type. The G3PDH protein deduced from G3PDH cDNA contains 699 amino acids, of which the molecular weight is 76.6 kDa and the isoelectric point is 6.49, with −3.33 charge in pH 7.0. This G3PDH gene and protein had 91% and 95% identity with the putative G3PDH gene sequence (AY845323.1) and protein (AAX56341.1) [20]. The putative G3PDH protein contained 701 amino acids, with the molecular weight of 76.9 kDa, isoelectric point of 6.1 and −6.08 charge in pH 7.0 environment. So, it was showed some certain structural differences between them, and also reflected the diversity and complicity of G3PDH isoenzyme with NAD^+^ as coenzyme. It was reported that three isoforms of G3PDH have been separated from *D. tertiolecta* [27], [28]. The chloroplasts contained the two major isoforms, and the third, minor form was in the cytosol. The first chloroplast form was the major form when the cells were grown on high NaCl, and it has been a form for glycerol production for osmoregulation. The second form increased in specific activity when inorganic phosphate was increased and played roles in stimulating cell growth and glyceride synthesis. The presumption of subcellular localization by WoLF PSORT showed that G3PDH in the present study may situate in the chloroplast as the osmoregulation form in glycerol production. Similarly, it was thought that the G3PDH in the study by He et al was also an osmoregulation form in chloroplast [20]. However, another study cloned and sequenced a cDNA encoding the *D. salina* FAD-G3PDH, which situated in the mitochondrial. The expression of FAD-G3PDH was enhanced by salt treatment. Its catalytic site facing toward the cytosol, combined action of this enzyme with the cytosolic NAD^+^-dependent G3PDH forms the glycerol-3-phosphate shuttle [29]. In this shuttle, cytosolic NAD^+^-dependent G3PDH oxidizes cytosolic NADH to NAD^+^, and catalyzes the reduction of DHAP to glycerol-3-phosphate. Subsequently glycerol-3-phosphate passes the outer mitochondrial membrane and is oxidized to DHAP by FAD-G3PDH, simultaneously delivers its electrons to the respiratory chain [30]. Due to the essential role in glycolytic pathway, G3PDH is one of the typically constitutive housekeeping genes in living organisms [31], [32]. Consistently, results of sequence alignment showed that G3PDH genes were conservative between plants and algae. Phylogenetic analysis indicated that G3PDHs of green algae clustered into one group. Difference of G3PDHs functions between plant and green algae will be interesting in coming study considering the unicellular green algae maybe more sensitive to the salinity of environmental conditions.

According to the analysis of conservative region of the G3PDH in the present study, it could be speculated that the G3PDH functional domain originate at the 331 amino acid of the amino sequence and the first 330 amino acids are potentially correlated with other properties of the protein. Namely, G3PDH protein has two independent functional domains, SerB and G3PDH domains. SerB (EC 3.1.3.3) and glycerol-3-phosphate phosphatase (EC 3.1.3.21) are attributed to the same type of hydrolase, and two enzymes have similar functions due to their active centers of similar size. Therefore, it was speculated the G3PDH might also have glycerol-3-phosphate phosphatase activity and can catalyze DHAP to glycerol directly without glycerol-3-phosphate phosphatase. Similarly, in the studies by He et al and He et al, protein domain analysis revealed that *DsGPDH2* in *D. salina* and *DvGPDH1* and *DvGPDH2* in *D. viridis* all encoded unique bi-domain proteins with C-terminal G3PDH domains and additional N-terminal SerB domains [20], [26]. It has been reported that such bi-domain G3PDHs only exist in green alga, but not in higher plants or other species, such as yeasts and animals [26]. For example, only one catalytic domain has been found exist in polypeptide chain of G3PDHs in yeasts *Debaryomyces hansenii*, *Candida glycerinogenes* and *Candidamagnoliae* [33]–[35]. The existence of unique bi-domain G3PDHs in these green algae might be the evolutionary consequence, which maintained a unique osmoregulation mechanism in green algae for survival in severe environments [26].

Some key enzyme genes related to glycerol metabolism, such as the cDNA of fructose-1, 6-diphosphate aldolase (DsALDP) and NAD^+^-G3PDH are cloned from *D. salina*. These genes have been transferred into bacteria or plants to increase the salt-tolerance of these species. Zhang et al transferred the DsALDP gene into *E. coli* cultured in media with different NaCl concentration to analyze its expression [36]. As a result, the bacteria expressing DsALDP exhibited a higher salt tolerance with increasing NaCl concentration than bacteria with no DsALDP expression. Moreover, Zhang et al transferred the DsALDP gene into tobacco by *Agrobacterium tumefaciens,* and DsALDP gene was expressed effectively in transgenic tobacco, which exhibited a higher salt tolerance [37]. In another report, a G3PDH gene from *D*. *salina* has been transferred into led discs cells of tobacco. RT-PCR analysis showed that G3PDH gene integrated into tobacco genome has produced mRNA [38]. In the present study, the prokaryotic expression vector pET-32a-G3pdh was constructed and transferred into *E. coli* strain BL21 (DE3). The analysis by SDS-PAGE showed that the G3PDH protein was expressed successfully in transgenic strains, and the further work would emphasize on transforming this G3PDH gene into other higher plants to improve their salt tolerance.

In conclusion, in the present research the cDNA of a NAD^+^-G3PDH was successfully isolated from *D. salina*. The cDNA was 2100 bp long, which encoded a deduced protein sequence of 699 amino acids with an estimated molecular weight of 76.6 kDa. Protein domain analysis revealed that G3PDH protein has two independent functional domains, SerB and G3PDH domains. The *D. salina* G3PDH was a nonsecretory protein that may be located in the chloroplast. The *D. salina* G3PDH had a closer relationship with *Dunaliella* G3PDHs than with those of other species in the phylogenetic analysis. The secondary and three-dimensional structure of the *D. salina* G3PDH is predicted. In addition, the prokaryotic expression vector pET-32a-G3pdh was constructed and transferred into *E. coli*, in which G3PDH protein was expressed successfully. To fully understand glycerol metabolism and osmotic adjustment based on glycerol in *Dunaliella*, future investigation should focus on the gene clone of some enzymes related to glycerol metabolism and the application of transgenic technology to increase salt-tolerance of other plants by transferring these cloned genes.
